# Association of Serum Vitamin B12 and Vitamin D Levels With Colorectal Cancer: A Cross-Sectional Study From Eastern India

**DOI:** 10.7759/cureus.109825

**Published:** 2026-05-28

**Authors:** Kakali Das, Ajit K Kushwaha, Mukesh Niraj, Bela Ekka

**Affiliations:** 1 Biochemistry, Rajendra Institute of Medical Sciences, Ranchi, IND; 2 Surgical Oncology, Rajendra Institute of Medical Sciences, Ranchi, IND

**Keywords:** carcinogen, chemiluminescent microparticle immunoassay (cmia), colorectal cancer, serum biomarkers, serum vitamin b12, vitamin d serum level

## Abstract

Background

Colorectal cancer (CRC) is one of the leading causes of cancer-related mortality worldwide, with increasing incidence in developing countries such as India due to changing dietary habits and sedentary lifestyles. Epigenetic alterations, particularly DNA methylation, play an important role in colorectal carcinogenesis and are influenced by micronutrients involved in one-carbon metabolism, such as vitamin B12. Vitamin D, on the other hand, regulates cell proliferation, apoptosis, differentiation, and immune responses through vitamin D receptor-mediated pathways. However, studies evaluating the association of serum vitamin B12 and vitamin D levels with CRC have shown inconsistent findings, especially in the Indian population. This study aimed to assess the association of serum vitamin B12 and vitamin D levels with newly diagnosed, treatment-naïve CRC patients in a tertiary care center in eastern India.

Materials and methods

This was a cross-sectional analytical study conducted in the Departments of Biochemistry and Surgical Oncology at Rajendra Institute of Medical Sciences, Ranchi, Jharkhand, India, from January 2024 to February 2025 after obtaining Institutional Ethics Committee approval. A total of 244 participants were enrolled, including 122 newly diagnosed, histopathologically confirmed CRC patients and 122 age- and sex-matched healthy controls. Serum vitamin B12 and 25-hydroxyvitamin D levels were measured using an automated chemiluminescent microparticle immunoassay (CMIA) system. Data were analyzed using the Mann-Whitney U test, chi-square test, and logistic regression analysis, with p < 0.05 considered statistically significant.

Results

There was no statistically significant difference in age or gender distribution between cases and controls. Serum vitamin B12 levels were significantly higher in CRC patients compared to controls (median 579 pg/mL vs. 367.5 pg/mL; p < 0.001). Around 39.3% of CRC patients had high vitamin B12 levels (>883 pg/mL), compared to 5.7% of controls. Conversely, serum vitamin D levels were significantly lower in CRC patients (median 17.95 ng/mL vs. 21.85 ng/mL; p < 0.001), with 60.7% of cases showing vitamin D deficiency compared to 42.6% of controls. Logistic regression analysis demonstrated a statistically significant positive association between vitamin B12 and CRC (OR = 1.001; p < 0.001) and a significant inverse association between vitamin D and CRC (OR = 0.953; p < 0.001).

Conclusion

Our study demonstrated significantly elevated serum vitamin B12 levels and significantly reduced vitamin D levels in CRC patients compared to healthy individuals. Vitamin B12 showed a weak positive association, whereas vitamin D demonstrated a significant inverse association with CRC, suggesting a possible protective role of vitamin D in colorectal carcinogenesis. These findings highlight the potential role of these micronutrients as biomarkers in CRC; however, further large-scale longitudinal studies are required to establish causality and clinical applicability.

## Introduction

Colorectal cancer (CRC) is one of the most common cancers worldwide and continues to be an important cause of cancer-related deaths. Worldwide, around 1.9 million new cases were diagnosed in 2020, and this number is expected to rise to 3.2 million by the year 2040 [1,2]. CRC was historically a disease of developed countries; however, the incidence of CRC is rising rapidly in developing countries such as India due to changes in dietary habits, lifestyle, and increasing sedentary behavior [3,4]. CRC was the third most common cancer in both urban and rural Indian populations and ranked third in terms of mortality; thus, it is important to study modifiable risk factors and useful disease markers for disease progression [4,5].

CRC is a heterogeneous disease with a complex genetic and epigenetic background. DNA methylation, for example, plays a vital role in controlling gene expression and maintaining genomic stability [6,7]. This highlights the importance of micronutrients, including vitamin B12, in processes involved in one-carbon metabolism [8]. Vitamin B12 plays an important cofactor role in one-carbon metabolism, where it serves to generate S-adenosylmethionine, the main methyl donor in the plethora of DNA methylation reactions [9]. Vitamin B12 deficiency can lead to widespread DNA hypomethylation and genomic instability, but elevated levels have also been reported in cancers, highlighting a complicated role in cancer biology [10,11].

Conversely, vitamin D has more than one biological function; it regulates cell proliferation, apoptosis, and differentiation, as well as modulation of the immune response [12]. The biologically active form of vitamin D binds to nuclear vitamin D receptors as a transcriptional regulator, affecting gene transcription related to tumor suppression, as well as key pathways in colorectal carcinogenesis (e.g., Wnt/β-catenin signaling) [13]. Epidemiological data investigating the association between these vitamins and the risk of CRC are contradictory. The relationship between circulating vitamin D and CRC risk has been extensively studied, with protective associations reported in multiple subsets of studies, but non-significant correlations have also been found [14]. Similarly, inconclusive findings have been presented in studies examining vitamin B12, including inverse associations, positive associations, and no independent relationship with CRC incidence [15].

While interest has increased in investigating the relationships between vitamin B12 and vitamin D, few studies have simultaneously assessed serum levels of these two vitamins among newly diagnosed, treatment-naïve CRC patients, especially in the Indian population. The objective of this study was to assess the association of vitamin B12 and vitamin D, either individually or in combination, with CRC in patients who had not received any prior treatment.

## Materials and methods

This hospital-based cross-sectional analytical study was conducted in the Departments of Biochemistry and Surgical Oncology over a duration of one year (January 2024 to February 2025) at a tertiary care teaching hospital in eastern India. All participants were informed, and written consent was obtained before initiation of the study. Ethical clearance was obtained from the Institutional Ethics Committee (memo no. 57; dated January 27, 2024) before initiation of the study.

A total of 244 participants were enrolled, including 122 cases with newly diagnosed CRC and 122 age- and sex-matched controls. Cases were defined as patients diagnosed with CRC, supported by histopathological confirmation, who had not undergone any treatment (surgery, chemotherapy, or radiotherapy) at the time of enrollment in the oncology department. The control group (CG) included apparently healthy individuals who accompanied patients to the general medicine outpatient department, had no previous history of malignancy or anemia, and did not have suspicious symptoms of colorectal pathology.

Participants who had taken vitamin supplements or had a malignancy within the previous two years were excluded from either group to minimize confounding. The sample size was calculated using a prevalence-based formula, assuming a five-year prevalence of CRC in India of 87 per 100,000 population, a confidence level of 95%, and a precision of 5%, which yielded a minimum required sample size of 122 participants per group [4].

Information on demographic data, clinical history, and lifestyle was collected through a structured questionnaire (see Appendix 1). Venous blood samples (3 mL) were collected from all participants after a minimum fasting period of 12 hours, ensuring the absence of hemolysis. Serum was isolated by allowing the samples to stand at room temperature, followed by centrifugation at 3000 rpm for 10 min. Serum vitamin B12 and 25-hydroxyvitamin D (25(OH)D) levels were measured on the same day using an automated chemiluminescent microparticle immunoassay (CMIA) system (Abbott ARCHITECT i1000SR, Abbott Laboratories, Abbott Park, IL, USA).

Vitamin B12 levels were classified as low (<187 pg/mL), normal (187-883 pg/mL), or high (>883 pg/mL); vitamin D status was classified as deficient (<20 ng/mL), insufficient (20-<30 ng/mL), or sufficient (≥30 ng/mL).

Data were entered into Microsoft Excel (Microsoft® Corp., Redmond, WA, USA) and analyzed using IBM SPSS Statistics for Windows, Version 27 (released 2019; IBM Corp., Armonk, NY, USA). Continuous variables were expressed as median and interquartile range (IQR) because of their non-normal distribution, and group comparisons were performed using the Mann-Whitney U test. Categorical variables were described as frequencies and percentages and were compared using the chi-square test. Logistic regression analyses were performed to compare serum vitamin levels with CRC, and odds ratios (ORs) and p-values were reported. Statistical significance was defined as a p-value of <0.05. No further multivariable adjustment was conducted beyond logistic regression analysis. Potential confounding factors, including dietary intake, BMI, and sunlight exposure, were not assessed in the study.

## Results

The sample size of the study was 244 participants, including 122 newly diagnosed CRC patients and 122 age- and sex-matched healthy individuals. Table 1 presents the baseline characteristics of the study population.

**Table 1:** Baseline Characteristics of Study Participants *Mann-Whitney U test; #Chi-square test.

Variable	Cases (n = 122)	Controls (n = 122)	Test Statistic	p-value
Age (years), Median (IQR)	49.0 (25.5-72.5)	49.0 (25.5-72.5)	6569.5	0.741^*^
Minimum Age	16	21	-	-
Maximum Age	82	83	-	-
Male, n (%)	71 (58.2%)	69 (56.6%)	0.067	0.797^#^
Female, n (%)	51 (41.8%)	53 (43.4%)	-	-
Vitamin B12 (pg/mL), Median (IQR)	579.0 (25.0-1630.3)	367.5 (210.6-524.4)	4659.0	<0.001*
Vitamin D (ng/mL), Median (IQR)	17.95 (11.9-24.0)	21.85 (14.2-29.6)	9505.5	<0.001*

**Figure 1:**
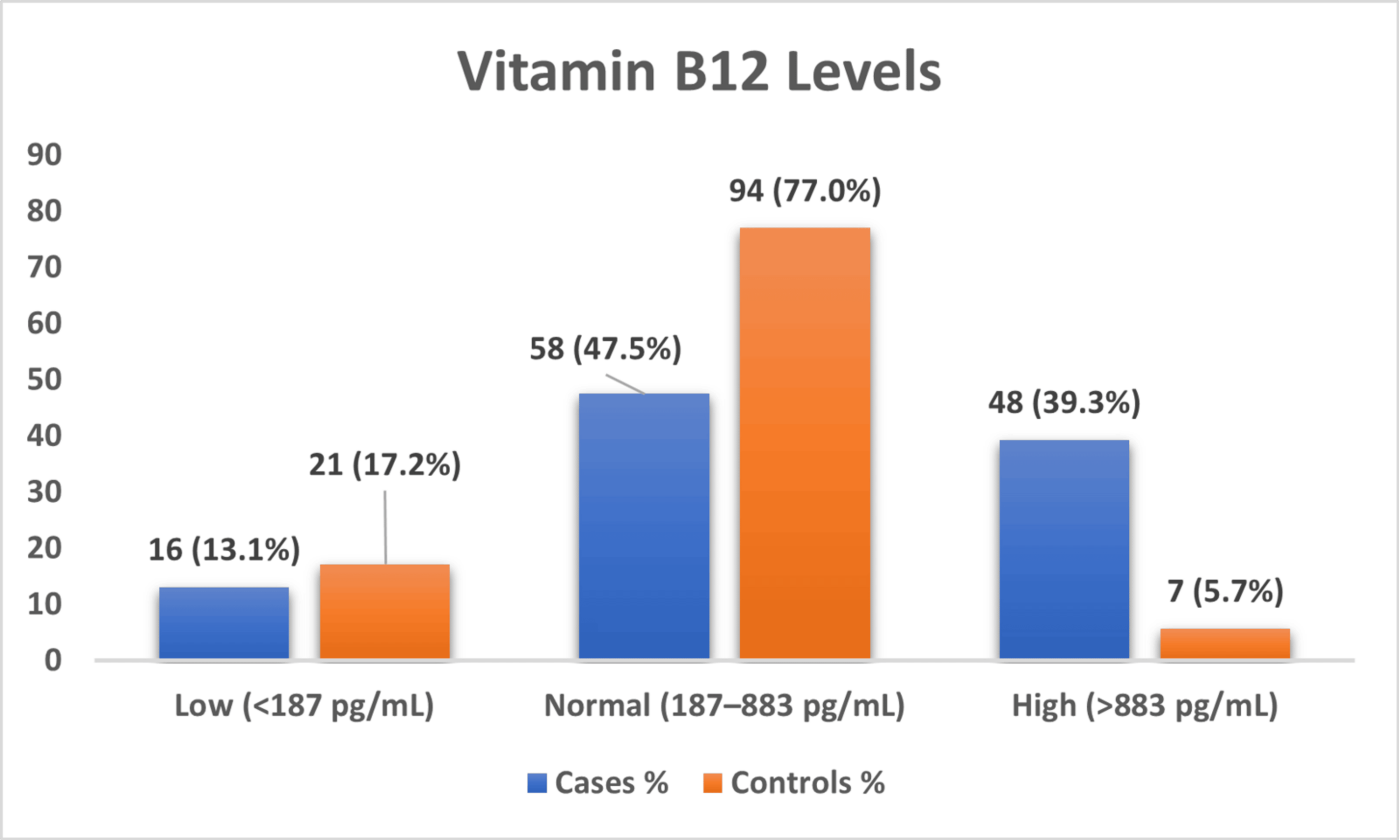
Distribution of Vitamin B12 Levels in Cases and Controls

There was no statistically significant difference in age between cases and controls (49.0 vs. 49.5 years; p = 0.741) or in gender distribution (p = 0.797). CRC patients had significantly higher serum vitamin B12 levels compared with healthy controls. The median vitamin B12 level in cases was 579 pg/mL (IQR, 1605.25), whereas in controls, it was 367.5 pg/mL (IQR, 313.75), and this difference was statistically significant (p < 0.001). A greater percentage of patients with CRC showed high vitamin B12 levels (>883 pg/mL) compared with controls (39.3% vs. 5.7%), whereas normal levels were more common among healthy individuals (77.0% vs. 47.5%) (Figure 1).

**Figure 2:**
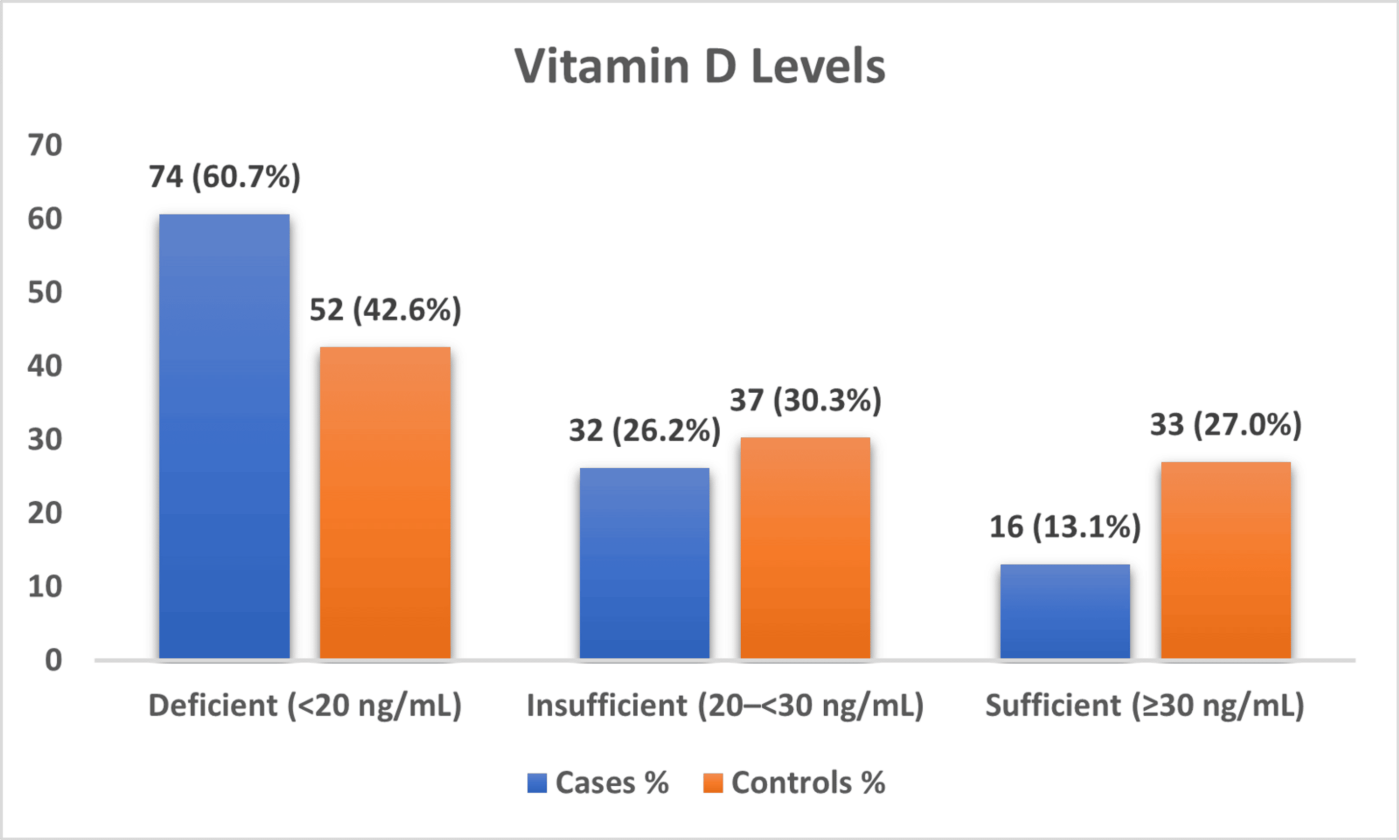
Distribution of Vitamin D Levels in Cases and Controls

Conversely, serum vitamin D levels were lower in CRC patients than in controls. The median vitamin D level was 17.95 ng/mL (IQR, 12.075) in cases and 21.85 ng/mL (IQR, 15.400) in controls (p < 0.001). CRC patients (60.7%) had a higher prevalence of vitamin D deficiency (<20 ng/mL) compared with controls (42.6%), and only 13.1% of patients, compared with 27.0% of controls, had adequate vitamin D levels (≥30 ng/mL) (Figure 2).

Logistic regression analysis assessing the association between serum vitamin levels and CRC showed a statistically significant positive relationship between serum vitamin B12 levels and CRC (β = 0.001, OR = 1.001; p < 0.001), as well as a statistically significant inverse association between vitamin D levels and CRC (β = -0.049, OR = 0.953; p < 0.001) (Table 2).

**Table 2:** Logistic Regression Analysis of Association with Colorectal Cancer

Variable	β Coefficient	Odds Ratio (OR)	p-value
Vitamin B12 (pg/mL)	0.001	1.001	<0.001
Vitamin D (ng/mL)	-0.049	0.953	<0.001

## Discussion

We investigated serum vitamin B12 and vitamin D levels, alone or jointly, in relation to CRC among treatment-naïve patients diagnosed with CRC. This study showed significantly higher vitamin B12 levels and significantly lower vitamin D levels in CRC patients than in healthy controls. There was a weak but statistically significant positive association for vitamin B12 (OR = 1.001; p < 0.001) and a significant inverse association for vitamin D in the logistic regression analysis (OR = 0.953; p < 0.001). The majority (39.55%) of cases were in the 46-55 years age group, and there was a slightly higher incidence in males (58.2%). These observations align with the epidemiological patterns presented by Abu Hassan et al., who observed the incidence of CRC to be greater in males across populations [16]. Similarly, Zhang et al. observed a comparable male predominance (~57%) in CRC cases, which is consistent with our findings [17]. While many studies show a peak incidence in older age groups, the younger age distribution observed in our study may suggest a rising trend of early-onset CRC in developing countries.

The serum vitamin B12 levels observed in our study were markedly elevated (median, 579 pg/mL vs. 367.5 pg/mL in controls), which is supported by Haghighat et al., who demonstrated a positive association between serum vitamin B12 and CRC [11]. Similarly, Sottotetti et al. reported elevated vitamin B12 levels in cancer patients, especially in advanced cases [18]. Furthermore, Tsilidis et al. reported a small positive association between circulating vitamin B12 and CRC risk (OR: 1.12), which is directionally consistent with our findings, although the magnitude of association was smaller [19]. However, the association between vitamin B12 and CRC remains under debate. Harnack et al. found no independent association between dietary vitamin B12 and CRC [15]. Similarly, Arthur et al. found no clear association between B-vitamin intake and CRC [20]. In contrast, Huang et al. demonstrated an inverse correlation between dietary vitamin B12 intake and CRC risk, which may indicate a protective effect [21]. Discrepancies between dietary and/or circulating levels, variations in metabolic status, and the complex role of vitamin B12 in DNA methylation may account for these inconsistencies. However, the positive association between vitamin B12 and CRC observed in this study does not establish a causal relationship.

The results of our study showed that the prevalence of vitamin D deficiency was significantly higher in patients with CRC compared with controls, while fewer patients had sufficient serum levels. These findings are in line with multiple reports showing an inverse association between vitamin D and CRC. Weinstein et al. demonstrated that higher circulating levels of 25(OH)D were associated with a lower risk of CRC [14]. Chandler et al. also observed a strong inverse association between prediagnostic vitamin D levels and both CRC incidence and mortality [22]. Abbasnezhad et al. further supported this association by demonstrating a lower risk of CRC with higher vitamin D levels [23].

The biological plausibility of this association is well established. Vitamin D, in its active form (calcitriol), binds to the vitamin D receptor, regulates gene transcription, and affects cell proliferation, apoptosis, and angiogenesis [12]. It also modulates critical signaling pathways, such as Wnt/β-catenin, that influence CRC carcinogenesis [12]. However, not all studies are in agreement. Acikgoz et al. found no significant correlation between vitamin D status and CRC, and Luo and Li found no statistically significant association between vitamin D intake and CRC risk [24,25]. These differences may be due to variations in study populations, measurement methodologies, and adjustment for confounders such as UV exposure and dietary intake.

Our data support the growing evidence of a link between vitamin D insufficiency and CRC risk, in addition to a possible association between elevated vitamin B12 levels and this disease; however, the relationship may be complex. A unique strength of this study is the concurrent measurement of both micronutrients in untreated cases, highlighting the need for further longitudinal studies to elucidate their association and potential role in colorectal carcinogenesis. Strengths of this study include its analytical design, age- and sex-matched controls, treatment-naïve CRC patients at the time of sample collection, and standardization of biochemical estimation using CMIA methods. Despite statistically significant findings (p < 0.05), these limitations hinder causal interpretation because of the cross-sectional design and limit generalizability due to the single-center setting. Additionally, potential confounding factors, such as smoking, alcohol use, educational status, and metabolic comorbidities, were not assessed because of logistical constraints. Due to the small sample size in each subgroup, stratified analysis could not be performed, and the cross-sectional design cannot accurately assess long-term vitamin status.

## Conclusions

Our study suggests that serum vitamin B12 levels are significantly elevated, whereas vitamin D levels are significantly reduced, in patients with CRC compared with healthy controls. A statistically significant but weak positive relationship was observed between vitamin B12 and CRC, while a significant negative association was observed for vitamin D. Together, these findings suggest that vitamin D may play a protective role in colorectal carcinogenesis. The relationship, however, appears to be complex and perhaps not clinically relevant in the case of vitamin B12. Nevertheless, extensive longitudinal studies are necessary to validate causality and evaluate their application as biomarkers or therapeutic targets in CRC.

## Appendices

Appendix 1: Case record form

SECTION A: PARTICIPANT IDENTIFICATION

· Case (Newly Diagnosed CRC Patient)

· Control (Healthy Individual)

Date of Enrollment: ______________________

Registration Number: ______________________

SECTION B: SOCIODEMOGRAPHIC PROFILE

1. Name of Participant: ___________________________________

2. Age (years): __________

3. Sex: Male Female

4. Residential Address: ___________________________________

5. Area of Residence: Urban Rural Semi-urban

6. Educational Status: Illiterate Primary School Secondary School Higher Secondary Graduate Postgraduate

7. Occupation: ___________________________________

8. Monthly Family Income (Approx.): ______________________

9. Marital Status: Single Married Widowed Divorced/Separated

10. Mobile Number: ______________________

SECTION C: ANTHROPOMETRIC AND GENERAL HEALTH DETAILS

1. Height (cm): __________

2. Weight (kg): __________

3. Body Mass Index (BMI): __________

4. Blood Pressure: ____________

5. Comorbidities: Diabetes Mellitus Hypertension Dyslipidemia Thyroid Disorder Chronic Liver Disease Chronic Kidney Disease None Others: ______________________

6. Current Medications: ___________________________________

SECTION D: CLINICAL DETAILS (FOR CRC CASES)

1. Date of Diagnosis of CRC: _____________

2. Histopathological Confirmation Available: Yes No

3. Site of Tumor: Colon Rectum Rectosigmoid Others: ______________________

4. Presenting Complaints and Duration:

**Table 3:** Presenting Complaints and Duration

Symptom	Present	Duration
Bleeding per rectum		
Altered bowel habits		
Loose stool		
Constipation		
Abdominal pain		
Weight loss		
Features of intestinal obstruction		
Loss of appetite		
Fatigue		

5. Stage of Disease (if available): Stage I Stage II Stage III Stage IV

6. Treatment Status Prior to Enrollment: Treatment-naïve Surgery Chemotherapy Radiotherapy

SECTION E: SCREENING DETAILS (FOR Non-CRC)

1. History of Any Malignancy in Last 2 Years: Yes No

2. History of Anemia: Yes No

3. Symptoms Suggestive of Colorectal Cancer:

**Table 4:** Symptoms Suggestive of Colorectal Cancer

Symptom	Yes	No
Bleeding per rectum		
Increased frequency of loose stool		
Features of intestinal obstruction		
Chronic abdominal pain		
Unexplained weight loss		

4. Fit for Inclusion as Healthy Control: Yes No

SECTION F: VITAMIN SUPPLEMENTATION AND SUNLIGHT EXPOSURE

1. History of Vitamin B12 Supplementation in Last 24 Months: Yes No

2. History of Vitamin D Supplementation in Last 24 Months: Yes No

3. Multivitamin Intake: Yes No

SECTION G: LABORATORY INVESTIGATIONS

Sample Collection Details

1. Date of Blood Collection: ____________

2. Fasting Status: ≥12 hours fasting Non-fasting

3. Hemolysis Present: Yes No

Serum Vitamin B12 Estimation

1. Serum Vitamin B12 Level: __________

2. Vitamin B12 Category: Low (<187 pg/mL) Normal (187-883 pg/mL) High (>883 pg/mL)

Serum Vitamin D Estimation

1. Serum Vitamin D Level: __________

2. Vitamin D Category: Deficient (<20 ng/mL) Insufficient (20-<30 ng/mL) Sufficient (≥30 ng/mL)

SECTION H: FINAL STUDY CLASSIFICATION

Participant Included As: Case Control

Reason for Exclusion (if excluded): ___________________________
